# No direct effect of the -521 C/T polymorphism in the human dopamine D4 receptor gene promoter on transcriptional activity

**DOI:** 10.1186/1471-2199-7-18

**Published:** 2006-05-24

**Authors:** Eva Kereszturi, Orsolya Kiraly, Csaba Barta, Noemi Molnar, Maria Sasvari-Szekely, Zsolt Csapo

**Affiliations:** 1 Department of Medical Chemistry, Molecular Biology and Pathobiochemistry, Semmelweis University, Budapest, Hungary; 2 Biological Research Center of the Hungarian Academy of Sciences, Szeged, Hungary

## Abstract

**Background:**

The human dopamine D4 receptor (DRD4) gene has been studied extensively as a candidate gene for certain psychological traits and several behavioural and psychiatric disorders. Both the 5' regulatory region and the coding sequence contain a number of polymorphisms. The promoter variants have received particular attention in the past few years due to their possible role in the regulation of gene transcription. Previously, the -521C/T SNP was shown to influence promoter activity. The aim of this study is to perform an in-depth analysis of this effect in the context of various neural cell lines.

**Results:**

Endogenous mRNA expression of the DRD4 gene was demonstrated in two neuroblastoma (SK-N-F1, IMR32) and one retinoblastoma cell line (Y79) by RT-PCR. In addition, very low DRD4 mRNA levels were also detected in HeLa cells. The transcriptional activity of a series of 5' promoter deletion mutants was determined by transient transfection of luciferase reporter constructs. The activity profile of these promoter fragments was similar in each of the cell lines tested. The highest luciferase reporter activity was obtained with a construct containing promoter sequences between nucleotides -668 to -389, while a putative silencer region was localised spanning from nucleotide -1571 to -800. Surprisingly, the -521 C/T polymorphism had no significant effect on transcriptional activity of the reporter construct with the highest activity (-668 to -389) in any of the three cell lines tested.

**Conclusion:**

Our results do not confirm previous data assigning different transcriptional activities to the -521 C/T alleles of the human DRD4 promoter. Furthermore, these findings highlight the need for further characterization of the 5' regulatory region of the DRD4 gene and identification of additional functional promoter polymorphic sites, especially in the context of haplotype.

## Background

Dopamine, an important neurotransmitter in the brain, plays a major role in the control of motor functions and behavioral patterns via interacting with specific cell surface receptors. Dopamine receptors belong to the large family of G protein-coupled receptors and can be classified as D1-like (D1, D5) and D2-like (D2, D3, D4) subgroups based on their sequence homology and pharmacological characteristics [1]. The human dopamine D4 receptor (DRD4) gene was originally cloned by Van Tol et al. [2]. The 5' flanking region of the gene was characterized by Kamakura et al. [3] as a housekeeping gene-like promoter. The promoter lacks a TATA or CAAT box but several putative transcription factor binding sites and CpG islands have been identified. 5'-RACE analysis of DRD4 mRNA revealed multiple transcription initiation sites located between nucleotides -501 and -436, relative to the translational start site. The region between -591 and -123 was found to contain the minimal promoter and to confer tissue-specific expression on the DRD4 gene and a putative repressor element was identified between nucleotides -770 and -679. Although high DRD4 mRNA expression was detected in the limbic area and frontal cortex associated with emotional function, motivation and cognition, the highest mRNA level was found in the retina [4]. The DRD4 plays an important role in cognitive functions and it is a target of numerous antipsychotic drugs widely used in psychiatric disorders [5].

The 5' regulatory region and coding sequence of the DRD4 gene harbour a number of polymorphisms which may arise due to its telomeric location on chromosome 11. Promoter polymorphisms of the DRD4 gene, including the 120 base pair (bp) duplication and several SNPs (-521 C/T, -616 G/C SNP), have been extensively studied as possible risk factors of psychological traits as well as behavioural and psychiatric disorders. Despite the numerous polymorphic allele variants, only one mutation has been described in this region [6]. A 27 bp deleted sequence was identified only in heterozygotes and with an extremely low allele frequency. To assess the potential functional role of the 27 bp deletion, an *in silico *analysis was performed that revealed various transcription factor binding sites within this short region.

An association between attention deficit hyperactivity disorder (ADHD) and the long form of the 120 bp duplication polymorphism was confirmed by a number of groups [7-9], although contradictory results have been reported by others [10]. Interestingly, the short form of this promoter length polymorphism has been associated with novelty seeking, a human personality trait sharing common characteristics with ADHD [11]. Seaman et al. [12] identified several consensus transcription factor binding sites within the 120 bp repeat, and there is *in vitro *evidence for enhanced Sp1 binding capacity of the duplicated form [13]. The functional role of these genetic variants was studied recently employing *in vitro *luciferase reporter assays, demonstrating a lower transcriptional activity for the duplicated allele compared to the shorter variant [14].

Another well studied polymorphism in the DRD4 regulatory region is the -616 C/G SNP, which is thought to interact with the recently described -615 A/G variant in the adjacent position [15]. It has been suggested that the -616 C/G SNP results in the creation of an AP-2 transcription factor binding site [16]. An association of the -616 C/G SNP with ADHD [17], schizophrenia [18] and personality traits [19] have been reported in the literature but have not been confirmed by others. Although there are no association studies concerning the -1217G insertion/deletion polymorphism, its putative functional effect was examined by fMRI and a correlation was found between the insertion allelic variant and a greater activation in the anterior cingulate cortex, a brain area mediating conflict [20].

One of the most studied polymorphisms of the DRD4 promoter region is the -521 C/T SNP. An association between novelty seeking and the -521 CC genotype was first identified in a Japanese population [21], followed by a replication in a Caucasian (Hungarian) population [22], although negative results have also been reported [23,24]. A recent meta-analysis showed a weak but positive association between this polymorphism and novelty seeking [25]. Lakatos et al. [26] revealed an association between the infant disorganized attachment and the T allele of the -521 C/T SNP but only in combination with the 7-repeat form of the DRD4 exon III polymorphism. A family-based association approach demonstrating a low-rate transmission of the DRD4 7-repeat – -521 T haplotype in a large secure group of infants [27] supports this finding, although results to the contrary have also been published [28]. Furthermore, a positive association was detected between the -521 C allele and schizophrenia [29], a disorder with elevated DRD4 mRNA levels in the frontal cortex [30], although others failed to replicate this result [18]. A weak relationship between the -521 C/T SNP and ADHD has also been reported [17]. To date, only one study has been performed that reveals the functional effect of the -521 C/T SNP [29], using chloramphenicol acetyltransferase reporter assay with the minimal promoter previously defined in the Y79 retinoblastoma cell line. The results from these experiments showed that the T allele reduced promoter activity by 40% relative to the C allele.

In the present study, we attempted to replicate the possible transcriptional effect of the -521 C/T SNP using a similar *in vitro *reporter system in a number of neuroblastoma and retinoblastoma cell lines.

## Results

### DRD4 gene expression in neural and non-neural cell lines

**Figure 1:**
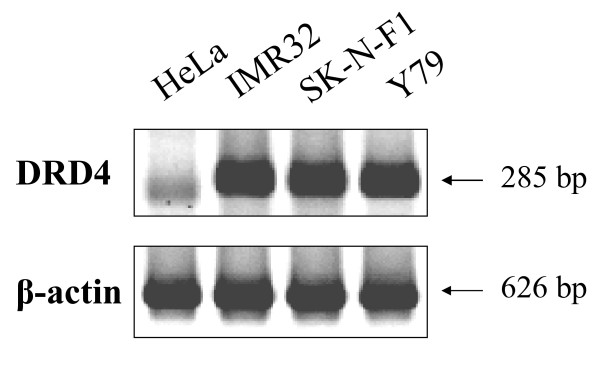
**Expression analysis of the DRD4 gene by RT-PCR**. β-actin mRNA levels within the same extracts were used as an internal control. Note that the primers for the DRD4 gene were designed to span the exon 2 and exon 3 boundaries, to control for possible genomic DNA contamination.

Previous studies suggested a wide range of putative regulator elements in the DRD4 promoter region that might be involved in gene expression. To better characterize the promoter, we performed luciferase assays in two neuroblastoma (SK-N-F1 and IMR32) and one retinoblastoma (Y79) cell line. A non-neural cell line (HeLa) was also included for comparison purposes. Prior to transcriptional analysis, the cell lines were analysed for endogenous DRD4 gene expression. Figure 1 shows the endogenous DRD4 mRNA levels in the four cell lines as detected by RT-PCR. Significant expression of the DRD4 gene was demonstrated in SK-N-F1 and IMR32 neuroblastoma cells, as well as in Y79 retinoblastoma cell line (Figure 1). A low but measurable band was also detected in HeLa cells, reflecting some DRD4 expression in this non-neural cell line previously observed by others [14]. There is an apparent difference in the mobility of the DRD4 transcripts in the HeLa and neural cell lines, likely resulting from the higher concentration of DRD4 transcripts in the neural cell lines. In other experiments in which much lower quantities of PCR product were loaded onto gels, no mobility shift was observed and only a very faint band was detected in case of HeLa (data not shown).

### DRD4 promoter deletional analysis in transiently transfected neural and non-neural cell lines

**Figure 2:**
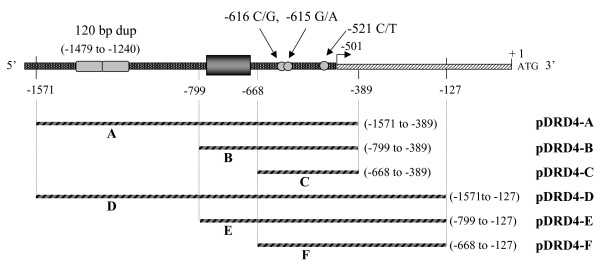
**Schematic representation depicting the location of the six 5' deletion mutant variants in the DRD4 gene promoter (pDRD4-A, B, C, D, E and F)**. The putative repressor region is indicated by a shaded box. The localization of several important SNPs and the 120 bp duplication region are also shown. The arrow at -501 denotes the transcription start site.

To verify whether the region upstream of the transcription start site has a repressor element between -770 and -679 – as previously suggested by Kamakura et al. [3], a panel of six 5' deletion mutants were created and cloned into the pGL3 promoterless vector, upstream of the luciferase reporter gene (Figure 2). Fragment A contained an 1183 bp sequence of the promoter region (-1571 to -389), including the 120 bp duplication polymorphism. Fragment B (-799 to -389) retained the putative negative modulator element, while fragment C (-668 to -389) lacked this element. Fragments D, E and F had the same 5' sequence as A, B and C, respectively, but included an extra 5' end region down to position -127. Fragments D, E and F were similar to those used in the Japanese study [3] with respect to the 5' UTR content. In all experiments, the promoterless pGL3-Basic vector served as a negative control and pTK-luc, a herpes simplex virus thymidine kinase promoter-containing vector, was used as a positive control.

Table 1 shows the relative luciferase activities of the different constructs normalized to β-galactosidase activity. Importantly, the pDRD4-B and C constructs exhibited the highest reporter gene expression levels among the DRD4 promoter constructs in each cell line. By contrast, the relative ratios of the promoter activity of the pDRD4 constructs relative to the pTK-luc strong promoter-containing construct, was much lower in HeLa cells than in cell lines of neural origin. These data are in good agreement with the results shown in Figure 1, indicating that endogenous DRD4 expression levels correlate well with the luciferase reporter activities.

**Figure 3:**
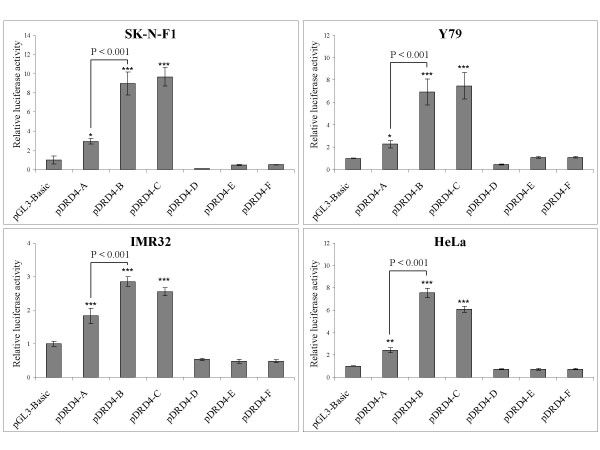
**The effect of 5' deletions on the transcriptional activity of the DRD4 promoter in four different cell lines**. Significant difference in promoter activity of pDRD4-A could be observed in each cell line relative to construct B (P < 0.001). Luciferase activity was normalized to the β-galactosidase activity. Data are expressed as fold increments over the pGL3-Basic activity and shown as mean ± SD. Results of a representative experiment are shown as measured in triplicates (*P < 0.05, **P < 0.01, ***P < 0.001 versus the corresponding promoterless vector in each cell type.) Similar data were obtained from three independent transfection experiments.

The six constructs used here exhibited similar expression patterns in each cell line (Figure 3). Surprisingly, the series of pDRD4-D, E and F constructs exhibited no significant transcriptional activity, while the pDRD4-A, B and C vectors showed elevated levels in relation to the promoterless vector (P < 0.05) (see Figure 3). Based on the data shown in Table 1 and Figure 3, there was no significant difference between the pDRD4-B and C vectors, which differed from one another only by the presence (B) or absence (C) of the putative repressor region. On the other hand, a significantly reduced promoter activity of pDRD4-A could be observed in each cell line when compared to construct B (P < 0.001) (see Figure 3). This suggests that the negative modulator sequence is located between positions -1571 and -800 as opposed to sequences -770 and -679, as proposed earlier [3]. Despite the moderate transcriptional activities of constructs pDRD4-D, E and F, their expression pattern was very similar to that of pDRD4-A, B and C respectively as measured in SK-N-F1 and Y79 cell lines. Namely, the constructs containing the putative repressor element located between positions -1571 and -800 (A and D) showed significantly reduced transcriptional activity compared to the vectors lacking this region (B, C and E, F, see Table 1).

### Effect of the -521 C/T SNP on transcriptional activity of DRD4 gene expression

**Figure 4:**
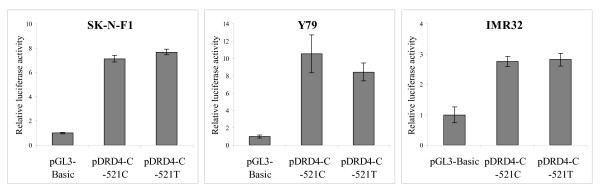
**The effect of the -521 C/T SNP on the transcriptional activity of the DRD4 minimal promoter in neural cell lines**. No significant difference could be detected between the transcriptional activities of the pDRD4-C constructs possessing the -521 C or T alleles in any of these cell lines. Luciferase activity was normalized to the β-galactosidase activity. Data are presented as fold increments over the pGL3-Basic activity and shown as mean ± SD. Results of a representative experiment are shown as measured in triplicates. Similar data were obtained from three independent transfection experiments.

For comparison of the relative promoter activity of the -521 C and -521 T alleles, the pDRD4-C construct was chosen as the parent plasmid since it exhibited the strongest transcriptional activity in all cell lines tested. After transient transfection of these DNAs into SK-N-F1, Y79 and IMR32 cells, a high level of luciferase activity was observed for each SNP construct relative to the promoterless pGL3 control vector (Figure 4). Nevertheless, under the conditions applied in these experiments, we could not detect any significant differences between the transcriptional activities of the pDRD4-C constructs possessing either the -521 C or T alleles in any of these cell lines.

## Discussion

Regulation of DRD4 gene expression is a significant issue, not only due to the well-known importance of this neurotransmitter receptor in brain function, but also because its highly polymorphic promoter has been implicated in a number of psychogenetic association studies. The human Y79 and IMR32 neuroblastoma cell lines reportedly express DRD4 mRNA [3]. In addition to these cells, another neuroblastoma cell line (SK-N-F1) was also shown to produce high level of DRD4 mRNA in our study (Figure 1). These results are in agreement with the expression pattern of the DRD4 mRNA described by Van Tol et al [2]. A low but detectable level of DRD4 mRNA was also observed in HeLa cells, indicating that the DRD4 promoter was slightly active in this cell line, in agreement with recent results [14], but in contrast to earlier data [3].

To characterize the DRD4 promoter, Kamakura et al. [3] performed CAT reporter assays using IMR32, Y79 and HeLa cell lines, and identified a putative promoter region between nucleotides -591 and -123 conferring tissue-specific DRD4 expression. In addition, a short region located 1.2 kb upstream from the initiation codon that harbours the 120 bp duplication, has also been shown to exhibit promoter activity [14]. Notably, we could not map any region with cell type-specific transcriptional activity in our experimental system, as the *in vitro *transcriptional activity of the six promoter constructs showed a similar expression pattern in all cell lines, including the non-neural HeLa cells (Figure 3). It is important to note, however, that a cell type-specific feature is often typical for members of the dopamine receptor family [31-36], indicating the need for additional studies that explore tissue-specific DRD4 expression in experimental systems that more closely mimic *in vivo *conditions.

In terms of transcriptional activity, our constructs could be classified into two main groups: the pDRD4-D, E and F group exhibited about 10 fold lower activity than the pDRD4-A, B and C group. In fact, plasmids pDRD4-D, E and F possessed similar or even lower activities than the promoterless pGL3-Basic vector (see Table 1). These results were quite surprising since the D, E and F fragments were very similar to those used in a former study [3]. A possible explanation for the inactivity of these promoter fragments could be due to the existence of four ATG codons residing with in this region (nucleotides -389 and -127) at positions -361, -346, -261 and -163. Three of these start codons are out of reading frame and could lead to the formation of a frame-shifted (inactive) luciferase gene. Another possibility is that a strong negative modulator element resides in this region.

The comparative analysis of constructs pDRD4-B (-799 to -389) and pDRD4-C (-668 to -389) was particularly interesting in light of a silencer region mapped to positions -770 and -679 by Kamakura et al. [3]. We observed no significant difference between the transcriptional activities of constructs with (pDRD4-B) or without (p-DRD4-C) the previously identified silencer region (see Figure 3 and Table 1). On the other hand, a significant difference was found between the *in vitro *transcriptional activity of pDRD4-B (-799 to -389) and pDRD4-A (-1571 to -389), suggesting the presence of a negative modulator in the region between positions -1571 and -800. Thus, the transcriptional activity data generated with constructs A, B and C has revealed yet another silencer region upstream from the one previously described [3]. Together, these results suggest that the human DRD4 gene promoter may possess two negative modulator regions, one of which did not function in our system. This hypothesized structure of the gene is reminiscent of that of the rat D2-, the Drosophila Dmdop1-, as well as the rat D3 dopamine receptor gene structure, which all contain two inhibitory elements in their promoter [32,37,38]. Nevertheless, the dopamine receptor promoters also show remarkable heterogeneity in their 5'-untranslated region. For example, the Drosophila Dmdop1 and the rat D2 dopamine receptor promoter contain an additional activating region beside the silencing elements [35,37]. Similarly, the human D1A and D5 dopamine receptor genes also comprise varying numbers of positive and negative modulator elements [31,34]. Beside the regulatory elements, the precise boundaries of the DRD4 promoter remain poorly defined. One study suggested that the promoter region might be within an intronic region [39]. This theory was based on previously reported discrepancies in the size of the DRD4 mRNA which was originally reported as 5.3 kb in the SK-N-MC human neuroblastoma cell line and in the striatum, medulla, frontal cortex and limbic area of the rat and monkey brain [2]. By contrast, the DRD4 mRNA was later found to be much shorter (1.5 kb), and expressed at higher levels, in the corpus callosum, medulla, subthalamic nucleus, as well as in the mesolimbic system and related regions. Furthermore, the DRD4 mRNA was barely detected in the striatum and the cerebral cortex [40] in contrast to the previous findings [2]. These observed differences in mRNA size suggest that the DRD4 gene might have alternative transcription start sites, as in the case of the catechol-O-methyltransferase (COMT) gene, resulting in the formation of two distinct transcripts with differing 5' ends [41]. Additionally, Todd and O'Malley suggested the possibility that a large intron might separate the coding exons of the DRD4 gene from the untranslated exons [39], as described for the dopamine D2 [35,36,42] and D3 [32] receptors. In conclusion, the 5' untranslated region of the dopamine D4 receptor shows a high degree of complexity, emphasizing the need for further characterization to better define the core promoter and transcription initiation site.

Another aim of our study was to corroborate the effect of the -521 C/T SNP on the transcriptional activity previously described by Okuyama et al. [21,29] however, in our system we observed no significant difference between the activities of the -521 C and -521 T alleles, in either the neuroblastoma or retinoblastoma cell lines (Figure 4). There were several differences in experimental setup between the previous and the present study, such as applying different reporter vectors and some variation in the length of the promoter fragment. The length of the cloned promoter fragments especially might have a significant effect on transcriptional activity [43]. An additional possibility for these conflicting results is the highly polymorphic nature of this gene. Other identified polymorphisms in the DRD4 promoter region, such as the SNPs in positions -768, -616, -615, -603 and -600, may also influence promoter activity. For this reason, a novel approach for elucidating the effect of these various polymorphisms on DRD4 gene expression would be to perform a comparative analysis of the SNPs in combination in the context of different genetic haplotypes.

## Conclusion

The aim of this study was to evaluate the effect of the -521 C/T SNP on DRD4 promoter activity. Our results do not confirm previous data assigning different transcriptional activities to the -521 C/T alleles in any of the three neuronal cell lines tested. The transcriptional activity of a series of 5' promoter deletion mutants was determined by luciferase reporter system. This experiments revealed a putative silencer region located between positions -1571 and -800.

## Methods

### Plasmid constructs

Six overlapping fragments were amplified by PCR from the promoter of the DRD4 gene using the following primers containing either an *Xho*I or an *Hind*III recognition site: DR4-S1 (5' acc a**ct cga g**tg ggc tgg act cgc cgt ttg gc 3', corresponding to region -1571/-1550; translation start site is referred to as position +1), DR4-S2 (5' aag g**ct cga g**cc cgg gac ccc ctg ccc agg 3', -799/-780), DR4-S3 (5' ggt t**ct cga g**aa ctg tgc aac ggg tgg ggc cg 3', -668/-647), DR4-AS1 (5' aag g**aa gct t**cc ctc ggg cgc tca ccc tag tcc 3', -389/-411), and DR4-AS2 (5' aag g**aa gct t**cc cgc ggc gct cca ccg tga 3', -127/-146). The reaction mixture contained 10 ng of genomic DNA, 1-1 μM of each primer, 1 U *Pfu *DNA polymerase (MBI Fermentas), 1x *Pfu *PCR buffer, 0.2 mM dNTP-mix, 1x solution Q (Qiagen) and the final MgSO_4 _concentration was set to 2.5 mM in a final volume of 10 μl. Amplification began with a 3 min denaturation step at 95°C followed by 35 cycles of 1 min denaturation at 94°C and 3 min annealing/synthesis at 72°C. A final extension step was applied at 72°C for 10 min. The amplified products were digested with *Hind*III and *Xho*I and cloned into the luciferase reporter plasmid pGL3-Basic (Promega). After transformation into *E. coli *DH5α, all the plasmids were purified with Qiagen Plasmid Midi Kit. Constructs were verified by sequencing. The -521 C/T alleles were generated using the QuickChange Site-Directed Mutagenesis Kit (Stratagene). The primers used were 5' ggc gtg gag gg**t**gcg cac gag gtc 3' (forward, -532/-509) and 5' gac ctc gtg cgc **a**cc ctc cac gcc 3' (reverse, -509/-532) for -521C site to T and 5' ggc gtg gag gg**c**gcg cac gag gtc 3' (forward, -532/-509) and 5' gac ctc gtg cgc **g**cc ctc cac gcc 3' (reverse, -509/-532) for -521T to C site. The mutations were confirmed by sequencing.

### Total RNA isolation, RT-PCR

Total RNA was isolated from neural and HeLa cells using the TRIzol reagent (Invitrogen) according to the manufacturer's procedure. The reaction contained 3 μg of total RNA, 0.2 μg random hexameric primers (Promega), 8 U RNasin (Promega) and DEPC-treated water in a final volume of 10 μl. The reaction mixture was heated to 70°C for 4 min, followed by the addition of 4 μl M-MuLV of reverse transcriptase 5x reaction buffer (Fermentas), 24 U RNasin, 0.5 mM dNTPs, 100 U RevertAid M-MuLV reverse transcriptase (Fermentas) and DEPC-treated water to final volume of 20 μl total. The reaction was incubated at 37°C for 40 min followed by 42°C for 20 min and 99°C for 5 min. Single-stranded cDNAs were amplified by DRD4 specific primers DART-3 (5' gca ccg cct cca tct tca acc 3') and DART-4 (5' cgg aac gtg gcc cag tag agc 3') in a final volume of 10 μl containing 1 μl of RT reaction mix, 1 μM of each primer, 5 U HotStarTaq DNA-polymerase, 1x HotStar PCR buffer, 1x Q solution and 0.2 mM dNTPs (Qiagen). Thermocycling conditions were as follows: incubation at 95°C for 15 min followed by 32 cycles of 94°C for 1 min, 68°C for 1 min and 72°C for 1 min completed with a final extension step at 72°C for 10 min. As described earlier, human β-actin gene served as an internal control [44]. PCR products were electrophorised in a 2% agarose gel.

### Cell culture

The human neuroblastoma cell line IMR32 and HeLa cells were cultured in minimal essential medium Eagle (Gibco) supplemented with 10 % fetal bovine serum, 1% nonessential amino acids and 1% Na-pyruvate. The human retinoblastoma Y79 cells were maintained in RPMI 1640 medium (Sigma) supplemented with 20 % fetal bovine serum and 1% Na-pyruvate. SK-N-F1 (neuroblastoma) cells were grown in Dulbecco's modified Eagle's medium, High Glucose (Gibco) supplemented with 10 % fetal bovine serum and 1% nonessential amino acids. All cell lines were grown at 37°C with 5% CO_2_.

### Transient transfections

SK-N-F1 transfection was performed by the calcium phosphate-DNA coprecipitation method. A total of 1.2 × 10^6 ^cells were seeded 24 h prior to transfection in six-well plates. Equimolar amounts of reporter plasmids (~ 5 μg) together with 2.5 μg internal control plasmid pCMV-β-gal were introduced into the cells. The calcium phospate precipitate was washed out 14 h after transfection and the cells were harvested 36 hours later and lysed in 100 μl of 250 mM Tris-HCl pH 8.0. HeLa cells were transfected with Lipofectamine 2000 (Invitrogen). A mixture of 1.7–2.0 μg reporter construct and 0.2 μg pCMV-β-gal and 6 μl Lipofectamine 2000 was used to transfect 0.4 × 10^6 ^cells plated 24 h before transfection in six-well plates. Y79 and IMR32 transfections were performed in 12 well plates. Approximately 1.6 × 10^6 ^cells (Y79) or 1.35 × 10^6 ^cells (IMR32) were plated 24 h before transfection and transfected with equimolar amounts of reporter plasmids (1.7–2.0 μg), 1 μg pCMV-β-gal combined with 4 μl Lipofectamine Reagent and 4 μl Plus Reagent (Invitrogen). In the case of HeLa, Y79 and IMR32 cells, 1–1.2 ml fresh medium was added after 5 hours of incubation, and the cells were allowed to grow for 48 h before harvesting.

### Luciferase and β-galactosidase assays

Cells were extracted by three consecutive freeze-thaw cycles and subsequent centrifugation. Supernatants were used for enzyme activity determinations. Luciferase and β-galactosidase activities were detected using the Luciferase Assay System kit (Promega) and by ONPG (o-nitrophenyl-β-D-galactopyranoside) cleavage rate, respectively. Luminescence was measured using a Mithras LB940 multilabel reader (Berthold Technologies, Bad Wildbad, Germany). Luciferase promoter activities were normalized to β-galactosidase activities. Three paralels were used in all transfections and all experiments were performed in triplicate.

### Statistical analysis

Statistical analysis was performed with one-way ANOVA followed by the Tukey-Kramer Multiple Comparison Test (GraphPad InStat).

## Abbreviations

DRD4, dopamine D4 receptor

SNP, single nucleotide polymorphism

RT-PCR, reverse transcriptase polymerase chain reaction

5'- RACE, 5' rapid amplification of cDNA ends

ADHD, attention deficit hyperactivity disorder

5' UTR, 5' untranslated region

## Authors' contributions

EK, OK and ZC performed the majority of the experimental work, CB participated in the luciferase assays and in the manuscript preparation, NM performed assays with the SK-N-F1 neuroblastoma cell line. MS participated in the design and coordination of the study, helped to evaluate the results and to draft the manuscript. All authors read and approved the manuscript.

## Figures and Tables

**Table 1:** Relative luciferase activity of the DRD4 promoter constructs. Luciferase activities of the crude cell extracts were normalized to their β-galactosidase activities. Data are shown as the mean ± SD. Results of a representative experiment are shown as measured in triplicates. Similar data were obtained from three independent transfection experiments.

**Luciferase/β-gal ratio**	**SK-N-F1**	**Y79**	**IMR32**	**HeLa**
pGL3-Basic	3.52 ± 1.43	3.87 ± 0.09	3.70 ± 0.29	3.59 ± 0.13
pDRD4-A	10.35 ± 1.00	8.80 ± 1.29	6.77 ± 0.86	8.71 ± 0.78
pDRD4-B	31.63 ± 4.7	26.89 ± 4.51	10.56 ± 0.52	27.08 ± 1.54
pDRD4-C	34.09 ± 3.34	28.98 ± 4.61	9.45 ± 0.46	21.89 ± 0.99
pDRD4-D	0.42 ± 0.02	1.75 ± 0.15	1.97 ± 0.12	2.56 ± 0.14
pDRD4-E	1.66 ± 0.20	4.18 ± 0.38	1.76 ± 0.22	2.51 ± 0.31
pDRD4-F	1.75 ± 0.17	4.21 ± 0.23	1.77 ± 0.16	2.63 ± 0.30
pTK-luc	84.21 ± 10.76	114.97 ± 10.50	28.73 ± 2.29	172.35 ± 2.05
